# The dual targeting ability of type II NAD(P)H dehydrogenases arose early in land plant evolution

**DOI:** 10.1186/1471-2229-13-100

**Published:** 2013-07-10

**Authors:** Lin Xu, Simon R Law, Monika W Murcha, James Whelan, Chris Carrie

**Affiliations:** 1 ARC Centre of Excellence in Plant Energy Biology, Bayliss Building M316 University of Western Australia, 35 Stirling Highway, Crawley, 6009, Western Australia; 2 Department of Biology I, Botany, Ludwig-Maximilians Universität München, Großhaderner Strasse 2-4, Planegg-Martinsried, D-82152, Germany

**Keywords:** Type II NAD(P)H dehydrogenases, Dual targeting, Mitochondria, Peroxisomes, Plastids

## Abstract

**Background:**

Type II NAD(PH) dehydrogenases are located on the inner mitochondrial membrane of plants, fungi, protists and some primitive animals. However, recent observations have been made which identify several Arabidopsis type II dehydrogenases as dual targeted proteins. Targeting either mitochondria and peroxisomes or mitochondria and chloroplasts.

**Results:**

Members of the ND protein family were identified in various plant species. Phylogenetic analyses and subcellular targeting predictions were carried out for all proteins. All ND proteins from three model plant species Arabidopsis, rice and Physcomitrella were cloned as N- and C-terminal GFP fusions and subcellular localisations were determined. Dual targeting of plant type II dehydrogenases was observed to have evolved early in plant evolution and to be widespread throughout different plant species. In all three species tested dual targeting to both mitochondria and peroxisomes was found for at least one NDA and NDB type protein. In addition two NDB type proteins from Physcomitrella were also found to target chloroplasts. The dual targeting of NDC type proteins was found to have evolved later in plant evolution.

**Conclusions:**

The functions of type II dehydrogenases within plant cells will have to be re-evaluated in light of this newly identified subcellular targeting information.

## Background

Eukaryotic cells are defined by the presence of subcellular compartments termed organelles, the presence of which allows for the partitioning of various biochemical pathways out of the cytosolic environment. Each organelle contains a specific complement of proteins that carry out specialised roles within the cellular landscape. However, while it was thought that specific proteins were only targeted to a single organelle, called location specific proteins, a large number of proteins have now been identified to be targeted to more than one organelle, and as such are termed dual targeted proteins. In plants, over 250 proteins have now been identified as dual targeted (Additional file 1) [1,2], the majority of which are targeted to both mitochondria and plastids. However, in recent years dual targeting of proteins has also been shown to occur between a number of additional organelles including: mitochondria and peroxisomes [1,3-7], plastids and peroxisomes [8-10], plastids and nucleus [11], plastids and endoplasmic reticulum [12], mitochondria and nucleus [3,13,14], cytoskeleton and peroxisomes [15], mitochondria and the cytoplasm [16], and plastids and the cytoplasm [17].

Mitochondrial respiration is an essential feature of plant metabolism, resulting in the generation of ATP in the process of oxidative phosphorylation. In addition to the classical electron transport chain coupled to phosphorylation, plant mitochondria contain a non-phosphorylating pathway. A major constituent of this alternative pathway is the type II or rotenone-insensitive NAD(P)H dehydrogenases (ND). Type II ND proteins were first identified in potato [18] and have also been identified in fungi, protists, some bacteria and some primitive animals [19]. The function of these type II ND proteins is that when they are linked to an alternative oxidase (AOX) they constitute a non-phosphorylating respiratory pathway that enables the redox status of the cell to evade adenylate control [20]. Specific studies altering the expression or inactivating the expression of specific type II ND proteins suggest roles in the capacity of NAD(P)H oxidation (*nda1*) [21]; reactive oxygen species formation and altered phenotypes (*ndb4*) [19].

The subcellular localisations of plant type II ND proteins have been a topic of debate in recent years. Initial *in vivo* GFP studies involving the targeting signals fused to GFP from five of the Arabidopsis ND proteins identified all proteins to be located in mitochondria [22]. Independent analyses using *in vitro* import assays into isolated mitochondria determined that three ND proteins (NDB1, NDB2 and NDB4) are externally located on the inner mitochondrial membrane and three were defined as internal (NDA1, NDA2 and NDC1) [23]. The final protein, NDB3 could not be cloned, and was concluded to be a pseudogene [23], although recent transcriptome analysis during germination suggests that NDB3 is expressed very early in germination [24]. However, subsequent studies have identified that Arabidopsis ND proteins are in fact dual-targeted, NDA1, NDA2 and NDB1 were found to be dual targeted to mitochondria and peroxisomes [25], and NDC1, was determined to be targeted to mitochondria and plastids [25,26]. The different conclusions from these studies can be readily reconciled by the fact that initial studies only used C-terminal GFP tags with the first 50 to 100 N-terminal amino acids of the target proteins [22]. However to ensure that targeting signals are not deleted or blocked, both N- and C-terminal GFP tagging is necessary [9,25]. Also with dual targeted proteins it appears that the mature or passenger protein can influence targeting to mitochondria and chloroplasts [27] so full length coding sequences should be tested to determine full targeting ability [25].

While desirable to use two or more complementing methods to determine subcellular localization [28], this can be difficult with dual targeted proteins in a variety of plant systems, requiring isoform specific antibodies and high purity of organelles. These tools do not exist except for Arabidopsis proteins and organelles. Of the known dual targeted proteins in Arabidopsis only 29 percent have been verified by 2 or more approaches (Additional file 1, 46 out of 165 proteins). This number drops to 15 percent when we look at dual targeted proteins identified in both target organelles by proteomic techniques (Additional file 1, 24 out of 165 proteins). By far the best and most widely used technique to discover dual targeted proteins has been fluorescent tagging (92 percent, Additional file 1, 152 out of 165 proteins). To date, no case of dual targeting using GFP tagging has been shown to be an artifact, in terms that while a false-negative rate may exist, due to the nature of the constructs as outlined above, false-positives have not been reported to date. Furthermore when assessing targeting, prediction and phylogenetic comparisons provides additional avenues to assess targeting, and along with GFP tagging, can be used to determine targeting ability of proteins in a variety of plant species, where direct biochemical approaches with gene families are not feasible.

Given that inactivation of specific ND genes in Arabidopsis has led to altered phenotypes [19], it is important to gain a wider perspective on the location of ND proteins in a variety of plants, as function can only be correctly determined if location is known. In order to gain a better insight into the targeting of plant type II ND proteins a study was undertaken to determine the subcellular localisation(s) of all ND proteins from *Oryza sativa* (rice) and *Physcomitrella patens* (Physcomitrella), that spans 500 million years of plant evolution, Thus in order to obtain a phylogenetic view of the dual targeting of ND proteins, subcellular localizations were analysed using GFP fusion proteins.

## Results

### Phylogenetic analyses and subcellular targeting predictions

**Figure 1:**
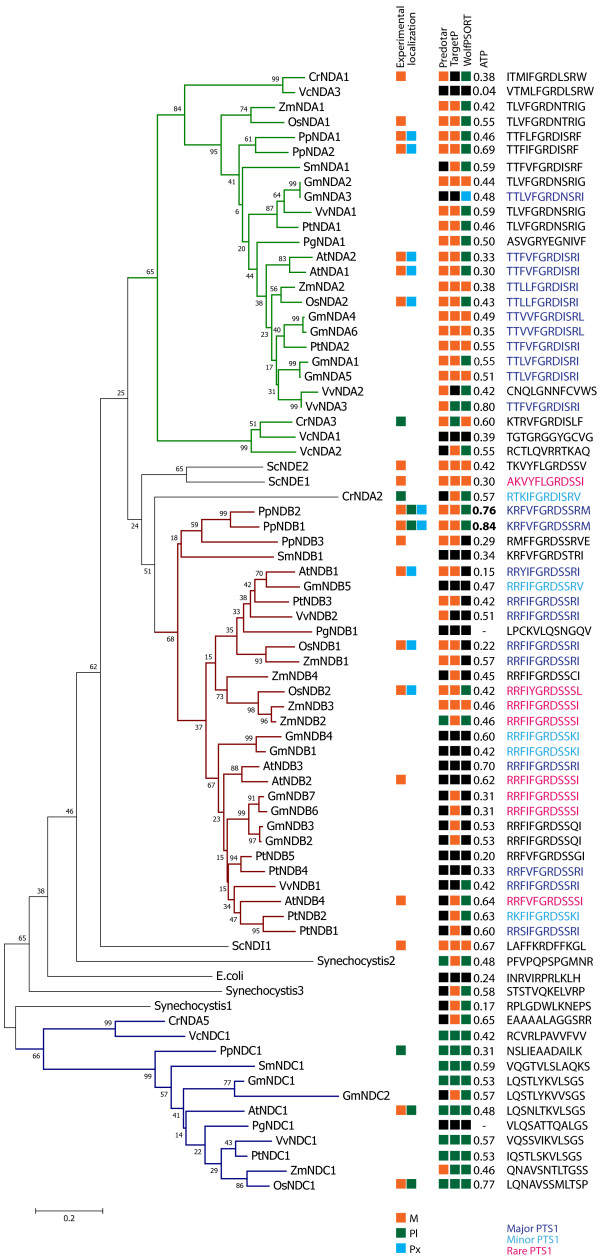
**Phylogenetic and subcellular predictions of plant NAD(P)H dehydrogenases.** Phylogenetic tree of NAD(P)H dehydrogenases from *Vovox carteri* (Vc)*, Chlamydomonas reinhardtii* (Cr), *Physcomitrella patens* (Pp), *Selaginella moellendorffii* (Sm), *Oryza sativa* (Os), *Zea mays* (Zm), *Vitis vinifera* (Vv), *Arabidopsis thaliana* (At), *Glycine Max* (Gm), *Picea glauca* (Pg) and *Populus trichocarpa* (Pt). The sequences of the *Saccharomyces cerevisiae* (Sc) NAD(P)H dehydrogenases are included as well as NADH dehydrogenases from *Synechocystis sp. PCC 6803* (Synechocystis) and *Escherichia coli* (E. coli). The predicted and experimental localizations of each protein (including this study) are indicated by the coloured boxes. Orange = Mitochondrial, Green = Plastid, Blue = Peroxisomal and Black = no prediction. Shown also are the last 12 amino acids (chosen because the PTS1 predictor uses the last 12 amino acids), putative peroxisomal targeting signals (PTS1) are coloured depending on the strength as determined by the AraPerox database (dark blue - major PTS1, light blue - minor PTS1 and pink - rare PTS1). The known experimental loactions for *Arabidopsis thaliana* ND proteins were taken from [23,25], for *Chlamydomonas reinhardtii* NDA1 and NDA2 from [32-34] and for *Saccharomyces cerevisiae* Ndi1, Nde1 and Nde2 from [35]. The phylogenetic tree was constructed using the neighboring-joining method. Numbers at each node are the percentage bootstrap values of 1000 replicates. The scale bar indicates the number of amino acid substitutions at each site.

Previously, we had determined that a number of ND proteins from *Arabidopsis thaliana* are dual targeted to several locations including: mitochondria and peroxisomes or mitochondria and plastids [25]. To gain a better understanding of how widespread this dual targeting ability is, we identified all the ND type proteins from a number of plant species, utilised various subcellular prediction programs for each ortholog and identified any peroxisomal type 1 targeting sequences (PTS1) according to several prediction programs [29,30] and the AraPerox database [31] (Figure 1 and Additional file 2). Phylogenetic analysis showed that all ND orthologs are clustered into three distinct clades (Figure 1). Types A, B and C (colored in green, red and blue respectively). It was found that A type ND proteins (in green) are predominantly predicted to be targeted to the mitochondria and is consistent with the previous data from Arabidopsis in which both NDA1 and 2 have cleavable mitochondrial N-terminal targeting signals [23]. However, it was interesting that at least one NDA ortholog from each higher plant species appears to have a PTS1 signal at their C-terminus. None of the prediction programs used predicted peroxisomal targeting for any of the NDA proteins from Physcomitrella (Pp) or Chlamydomonas (Cr).

Amongst the NDB type proteins, prediction of mitochondrial targeting was less frequent (Figure 1 and Additional file 2). This may be due to the fact that NDB proteins do not contain a cleavable N-terminus mitochondrial targeting signal which is the basis of the prediction programs used [23]. However, the majority (22 out of 29) of NDB proteins, ranging from Physcomitrella to Poplar also contain predicted PTS1 sequences. It is interesting to note that the predicted PTS1 sequences in both NDA and NDB proteins are not conserved in all species. In fact, in different species the PTS1 sequence is completely different in terms of the amino acids making up the last C-terminal tripeptide; however, the properties remain the same. This suggests that the C-terminal regions of NDA and NDB proteins is not crucial for catalytic function. The NDC type proteins, so named for their similarity to a cyanobacterial NADH dehydrogenase, and are mostly predicted to be targeted to plastids [22] (Figure 1). No PTS1 sequences were predicted. This is consistent with the localisation of Arabidopsis NDC1, which is predicted to be plastidial but has been experimentally shown to be targeted to both mitochondria and plastids [25,26].

### Subcellular targeting of NDA proteins

**Figure 2:**
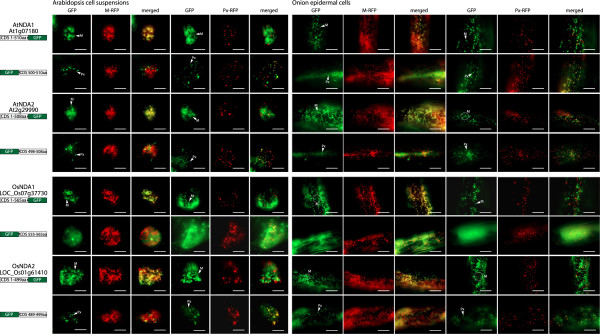
**Subcellular localizations of Arabidopsis and rice NDA type proteins.** GFP was fused to the C-terminus of NDA1 and NDA2 from *Arabidopsis thaliana* (At) and NDA1 and NDA2 from *Oryza sativa* (Os). Additionally, the last 10 amino acids were fused to the C-terminus of GFP. Subcellular targeting was analyzed in both Arabidopsis cell suspension or onion epidermal tissue along with RFP tagged mitochondrial or peroxisomal controls. The protein accession number, the location of GFP and the number of amino acids used are shown for each construct. Mitochondria (M) and peroxisomes (Px) are indicated. M-RFP - mitochondrial RFP, Px-RFP - peroxisomal RFP. Scale bar indicates 10 μm.

It has previously been shown that both NDA proteins from Arabidopsis (AtNDA1 and AtNDA2) are dual targeted to mitochondria and peroxisomes by both GFP-tagging and western blot analysis [25]. To demonstrate the fluorescence patterns expected by a dual targeted protein containing an N-terminal mitochondrial targeting signal and a C-terminal PTS1 sequence we have included the Arabidopsis proteins in our analysis of the rice and Physcomitrella NDA orthologs. When GFP is placed at the C-terminus of both AtNDA1 and 2, the chimeric protein is targeted to mitochondria in both Arabidopsis cell suspension and onion epidermal cells (Figure 2) as visualized by the overlap of the GFP with the RFP signals from the mitochondrial controls. In most instances the GFP signal shows a halo-like shape around the mitochondria. This has been speculated to result when the GFP is not completely pulled into the mitochondria and has previously been observed with AtNDA and AtNDB proteins [25] and is also observed with the GFP tagging of mitochondrial outer membrane proteins [36]. However in this case, as NDA proteins are known to be located in the inner membrane, it is possible that the C-terminus of NDA proteins are located in the intermembrane space (IMS) and may produce a similar result [4]. In contrast to the full-length C-terminal fusions, truncation of both AtNDA1 and AtNDA2 to just the final 10 amino acids fused to the C-terminus of GFP (10 amino acids were chosen to be consistent with previous research on ND targeting in Arabidopsis as well to be consistent with previous work on the targeting of peroxisomal proteins which also used the last 10 amino acids), was found to target solely to the peroxisomes (Figure 2). This co-localization was observed in both tissue types tested.

**Figure 3:**
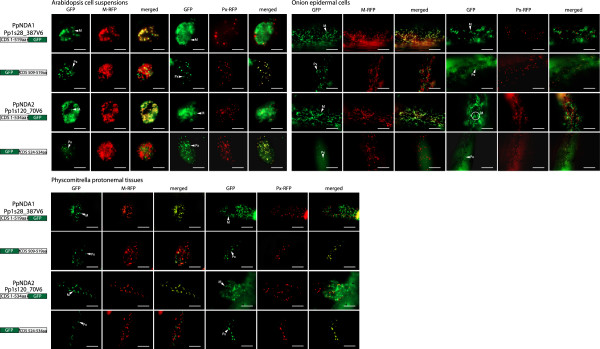
**Subcellular localizations of Physcomitrella NDA type proteins.** GFP was fused to the C-terminus of NDA1 and NDA2 from *Physcomitrella patens* (Pp). Additionally, the last 10 amino acids were fused at the C-terminus of GFP. Subcellular targeting was analyzed in both Arabidopsis cell suspension, onion epidermal tissue and Physcomitrella protonemal tissue along with RFP tagged mitochondrial or peroxisomal controls. The protein accession number, the location of GFP and the number of amino acids used are shown for each construct. Mitochondria (M) and peroxisomes (Px) are indicated. M-RFP - mitochondrial RFP, Px-RFP - peroxisomal RFP. Scale bar indicates 10 μm.

When both NDA proteins from rice (OsNDA1 and 2) were analysed in the same manner, slightly different results were obtained. OsNDA1 was observed to target to mitochondria when GFP was fused to its C-terminus (Figure 2), however when the last 10 amino acids are fused to the C-terminus of GFP, no targeting was observed to any distinct organelle (Figure 2), consistent with the fact that OsNDA1 does not contain a predicted PTS1 signal. When the targeting of OsNDA2 was tested it was seen to target to both mitochondria and peroxisomes in both the tissues tested (Figure 2). Therefore, at least one of the rice NDA proteins displays dual targeting ability. To determine if dual targeting ability is also evident with Physcomitrella NDA1 and NDA2, the same set of experiments were performed. In both onion epidermal cells and Arabidopsis cell suspension both Physcomitrella NDA proteins (PpNDA1 and PpNDA2) displayed dual targeting ability to both mitochondria and peroxisomes (Figure 3) as with AtNDA1, AtNDA2 and OsNDA2. However, based on the bioinfomatic analysis the targeting of PpNDA1 and PpNDA2 to peroxisomes is surprising as they do not contain any PTS1 signal nor are they predicted to be peroxisomal (Figure 1). To test whether this peroxisomal targeting is an artifact, we expressed the Physcomitrella NDA fusions in Physcomitrella tissue, thereby eliminating any incorrect targeting arising from cross-species interference. Again, both PpNDA1 and PpNDA2 were dual targeted to mitochondria and peroxisomes (Figure 3), suggesting that the dual targeting of NDA type proteins arose early in land plant evolution.

### Subcellular targeting of NDB proteins

**Figure 4:**
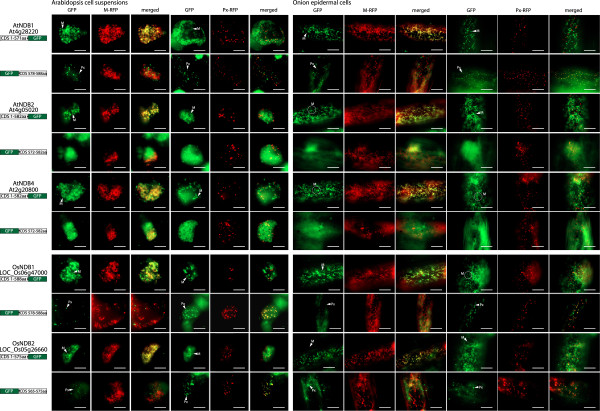
**Subcellular localizations of Arabidopsis and rice NDB type proteins.** GFP was fused to the C-terminus of NDB1, NDB2 and NDB4 from *Arabidopsis thaliana* (At) and NDB1 and NDB2 from *Oryza sativa* (Os). Additionally, the last 10 amino acids were fused at the C-terminus of GFP. Subcellular targeting was analyzed in both Arabidopsis cell suspension or onion epidermal tissue along with RFP tagged mitochondrial or peroxisomal controls. The protein accession number, the location of GFP and the number of amino acids used are shown for each construct. Mitochondria (M) and peroxisomes (Px) are indicated. M-RFP - mitochondrial RFP, Px-RFP - peroxisomal RFP. Scale bar indicates 10 μm.

The three Arabidopsis NDB proteins (AtNDB1, AtNDB2 and AtNDB4) have previously been determined to be mitochondrial [22,25], which was confirmed in this study (Figure 4). However, the last 10 amino acids of AtNDB1 fused to the C-terminus of GFP targeted to peroxisomes, whilst the last 10 amino acids of AtNDB2 and AtNDB4 did not target GFP to any distinct organelle and appeared cytosolic, indicative of no targeting (Figure 4). This confirms that AtNDB1 is dual targeted to both mitochondria and peroxisomes and that AtNDB2 and AtNDB4 can only be targeted to mitochondria. To determine if dual targeting of NDB proteins also occurs in rice, the two rice orthologs (OsNDB1 and OsNDB2) were also analysed. Both OsNDB1 and OsNDB2 were observed to target GFP to mitochondria and the last 10 amino acids can target GFP to peroxisomes in all the tissues tested (Figure 4). This suggests that the dual targeting of NDB proteins is conserved in rice and Arabidopsis.

**Figure 5:**
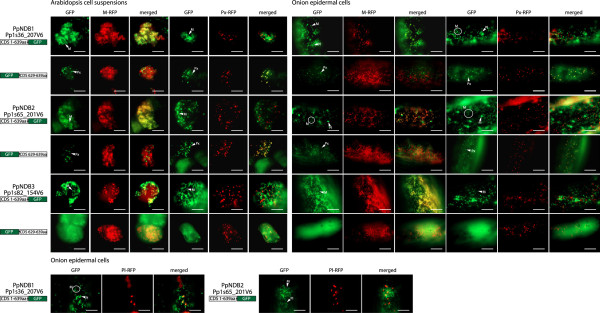
**Subcellular localizations of Physcomitrella NDB type proteins.** GFP was fused to the C-terminus of NDB1, NDB2 and NDB4 from *Physcomitrella patens* (Pp). Additionally, the last 10 amino acids were fused at the C-terminus of GFP. Subcellular targeting was analyzed in both Arabidopsis cell suspension or onion epidermal tissue along with RFP tagged mitochondrial, peroxisomal or plastid controls. The protein accession number, the location of GFP and the number of amino acids used are shown for each construct. Mitochondria (M), Plastids (Pl) and peroxisomes (Px) are indicated. M-RFP - mitochondrial RFP, Px-RFP - peroxisomal RFP, Pl-RFP - platid RFP. Scale bar indicates 10 μm.

**Figure 6:**
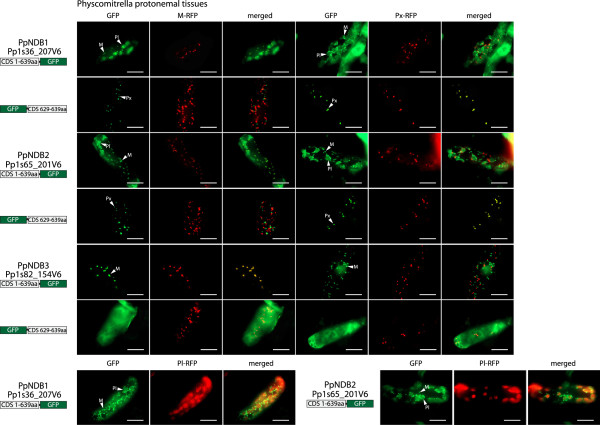
**Subcellular localizations of Physcomitrella NDB type proteins in Physcomitrella tissue.** GFP was fused to the C-terminus of NDB1, NDB2 and NDB4 from *Physcomitrella patens* (Pp). Additionally, the last 10 amino acids were fused at the C-terminus of GFP. Subcellular targeting was analyzed in Physcomitrella protonemal tissue along with RFP tagged mitochondrial, peroxisomal or plastid controls. The protein accession number, the location of GFP and the number of amino acids used are shown for each construct. Mitochondria (M), Plastids (Pl) and peroxisomes (Px) are indicated. M-RFP - mitochondrial RFP, Px-RFP - peroxisomal RFP, Pl-RFP - platid RFP. Scale bar indicates 10 μm.

Unlike rice and Arabidopsis, Physcomitrella contains three NDB homologs (PpNDB1, PpNDB2 and PpNDB3) and when GFP was fused to their C-termini, GFP was targeted to the mitochondria with a distinctive halo-like fluorescence in Arabidopsis cell suspensions (Figure 5). However, when these same constructs were expressed in onion epidermal cells a very different picture emerged for PpNDB1 and PpNDB2. Not only were PpNDB1 and PpNDB2 targeted to mitochondria but they could also target GFP to plastids (Figure 5). For PpNDB3, only mitochondrial targeting was observed (Figure 5). When the last 10 amino acids of PpNDB1, PpNDB2 and PpNDB3 were fused to the C-terminus of GFP, both PpNDB1 and PpNDB2 could target GFP to peroxisomes and PpNDB3 showed targeting of GFP to the cytosol (Figure 5). The GFP assays were repeated with Physcomitrella tissue (Figure 6) and as seen in the onion epidermal cells and Arabidopsis cell suspensions both PpNDB1 and PpNDB2 were able to target GFP to mitochondria, plastids and peroxisomes (Figure 6). PpNDB3 was only observed to target GFP to mitochondria, even in a homologous system (Figure 6). The lack of peroxisomal targeting by the C-termini of PpNDB3 is not surprising, as no PTS1 sequence is predicted (Figure 1). However, the targeting of PpNDB1 and PpNDB2 to plastids was unexpected. However, they are closely related to the Chlamydomonas NDA2 protein (Figure 1), which has been demonstrated to be a solely plastidic protein [32,33]. Notably, Physcomitrella NDB proteins are also considerably longer at the N-terminus compared to Arabidopsis (Additional file 3), suggesting additional targeting information and as the ambiguous targeting predictor (ATP) predicts both PpNDB1 and PpNDB2 to be dual targeted (Figure 1 and Additional file 2).

### Subcellular targeting of NDC proteins

**Figure 7:**
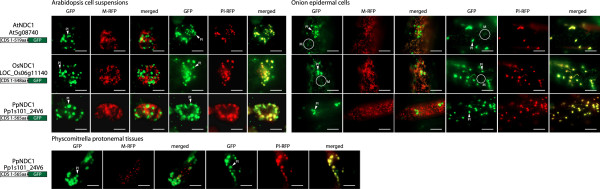
**Subcellular localizations of Physcomitrella NDC type proteins.** GFP was fused to the C terminus of the NDC type proteins from *Arabidopsis thaliana* (At), *Oryza sativa* (Os) and *Physcomitrella patens* (Pt). Subcellular targeting was analyzed in Arabidopsis cell suspension, onion epidermal tissues and for the Physcomitrella NDC1 also in Physcomitrella protenemal tissue along with RFP tagged mitochondrial or plastid controls The protein accession number, the position of GFP and the number of amino acids used are shown for each construct. Mitochondria (M) and plastids (Pl) are indicated. M-RFP - mitochondrial RFP, Pl-RFP - plastid RFP. Scale bar indicates 10 μm.

Phylogenetic analysis showed that among the three clades made up of NDA, NDB and NDC proteins, NDC is the most ancestral group and branched out early in evolution. With regard to the targeting ability of NDC proteins, only one ortholog was found in Arabidopsis, rice and Physcomitrella. When Arabidopsis NDC1 (AtNDC1) was fused to GFP and expressed, two different patterns emerged. In Arabidopsis cell suspensions a clear plastidic localization could be observed, however, in onion epidermal cells clear targeting to both mitochondria and plastids was observed (Figure 7). As AtNDC1 was previously shown to be imported into isolated Arabidopsis mitochondria [23,25,26] it can be concluded that AtNDC1 is dual targeted to mitochondria and plastids [25,26].

To determine when dual targeting of NDC1 arose we analysed the targeting ability of rice (OsNDC1) and Physcomitrella (PpNDC1). For OsNDC1 we see a similar pattern as with AtNDC1 in that plastid targeting was observed in Arabidopsis cell suspensions and dual targeted to mitochondria and plastids in onion epidermal cells (Figure 7). For PpNDC1 only plastid targeting was observed in both Arabidopsis suspension and onion epidermal cells (Figure 7). GFP tagging of PpNDC1 in Physcomitrella tissue resulted in only plastid targeting (Figure 7). These results suggest that in lower plants such as Physcomitrella, NDC1 proteins are only targeted to plastids, and in higher plants such as Arabidopsis and rice NDC1 has additionally acquired targeting to mitochondria during plant evolution.

## Discussion

This aim of this study was to determine the targeting ability of plant ND proteins in land plants. Aquatic green algae and land plants diverged over a billion years ago [37]. Examination of genes encoding ND proteins in Chlamydomonas revealed five genes. The closest NDA ortholog between Chlamydomonas and the higher plant NDA proteins is CrNDA1 (Figure 1). CrNDA1 has been identified as an inner mitochondrial protein, with roles in the oxidation of the mitochondrial matrix NADH in the absence of complex I [34]. In this study, it was observed that both PpNDA1 and PpNDA2 are dual targeted to mitochondria and peroxisomes (Figure 3), indicating that the dual targeting of NDA arose early in land plant evolution. Although the tripeptide SRF in PpNDA1 and PpNDA2 is not predicted to direct peroxisomal import (Additional file 2), it has been experimentally validated as a plant PTS1 tripeptide [30,38]. The contradiction of targeting between predictive and experimental data may be due to the fact that for non-canonical PTS1 tripeptides targeting ability depends largely on the upstream sequence [39]. The reason of prediction inaccuracy for non-canonical PTS1 proteins is that the proteins containing canonical PTS1 tripeptides dominate the dataset, most of which lack upstream enhancing sequences for targeting [30,39]. In Angiosperm plants such as Arabidopsis and rice, dual targeting of NDA proteins is not only conserved, but peroxisomal targeting is “strengthened” by the acquisition of canonical PTS1 targeing signals. In AtNDA1, AtNDA2 and OsNDA2, all contain the PTS1 tripeptide SRI; a feature of many proteins known to target to the peroxisomes [38]. In summation, while the acquisition of dual targeting ability in land plants cannot be dated precisely, it arose early and is conserved.

The closest NDB ortholog between Chlamydomonas and the higher plant NDB proteins is CrNDA2 (we have kept the name from previous publications however it clusters with NDB family (Figure 1)). It has been demonstrated that CrNDA2 was located within the thylakoid membranes of plastids and is crucial for nonphotochemical plastoquinone reduction and associated processes in Chlamydomonas [32,33]. The plastid localisation is interesting, as Physcomitrella NDB proteins (PpNDB1 and 2) are also targeted to plastids (Figures 5 and 6), in addition to mitochondria and peroxisomes. It is speculated that the Chlamydomonas and Physcomitrella proteins are able to target plastids because they contain an N-terminal extension, in contrast to the Arabidopsis and rice NDB proteins (Additional file 3). This extension ranges from 26 amino acids in Chlamydomonas and up to 60 amino acids in PpNDB1 and PpNDB2; which may carry extra targeting signals for plastids. In regards to the peroxisomal targeting of plant NDB proteins, it can also be hypothesized that this targeting ability arose very early in plant evolution. Analysis of the C-terminal end of CrNDA2 while not predicted to be peroxisomal, it does contain the tripeptide SRV which has been demonstrated to target peroxisomes previously [38]. However previous studies only used western blots on isolated plastids and mitochondria, and the testing of peroxisomes was not performed [32,33]. It is possible that even in Chlamydomonas, NDB type proteins contained peroxisomal targeting ability. In terms of plastid targeting, this ability has been lost in the higher plants of Arabidopsis and rice, possibly due to the loss of the N-terminal extension found in the Chlamydomonas and Physcomitrella sequences. A possible reason for this loss of plastid targeting is that in higher plants, plastids contain a type I ND which functions in non-photochemical plastoquinone reduction, however, such genes are absent from the genomes of many green algae including Chlamydomonas [40]. The situation in Physcomitrella would be the intermediate form, as Physcomitrella contain both the type I and type II ND proteins. This is consistent with plants starting out with only type II ND proteins in plastids followed by the gain of type I ND proteins which would facilitate the loss of the type II ND proteins. Therefore the role of NDB like proteins in the plastids of higher plants has become redundant.

The evolutionary history for NDC type proteins appears simpler as each plant genome encodes only a single copy of the gene. However the targeting ability of these proteins across the different model plants is not the same. Currently, there is no existing data on the targeting of Chlamydomonas CrNDA5, the closest homolog to NDC proteins in higher plants. Recently, the role of AtNDC1 in plastids has been analysed. AtNDC1 was found to be involved in the reduction of a plastoquinone analog *in vitro* and affect the overall redox state of the total plastoquinone pool *in vivo*[26]. AtNDC1 was also demonstrated to be required for normal platochromal-8 accumulation and is essential for viatmin K(1) production [26]. AtNDC1 has also been found to associate with the plastoglobules within plastids [41,42]. It is tempting to speculate that NDC1 proteins perform these same functions in all plant species but as yet no functional data for the role of NDC1 protein outside of Arabidopsis exists. However the fact that a NDC1 protein is found in all species analyzed (Figure 1) suggests that it does have a required function in plants. It appears that the targeting ability of NDC1 proteins and functional data for AtNDC1 support the hypothesis that the mitochondrial targeting of NDC1 proteins is a newly acquired phenomenon. However, the exact role for NDC1 proteins within the mitochondrial inner membrane has not yet been defined.

The dual targeting of plant ND proteins to mitochondria (and chloroplasts) and peroxisomes appears to have arisen early within the evolution of plants. We cannot at this time date this accurately, as we cannot rule out the possibility of the NDA and NDB type proteins from *Chlamydomonas reinhardtii* being targeted to peroxisomes. Based on the results presented on the targeting of ND proteins to peroxisome from Physcomitrella, the lack of a predicted PTS1 sequence is not reliable enough to say that ND proteins are not targeted to peroxisomes. Thus ND proteins from *Chlamydomonas reinhardtii* may also be targeted to peroxisomes.

Various studies have been undertaken to try and determine the cellular roles of type II ND proteins in higher plants. By utilising over expression and inactivation of different ND proteins it has been demonstrated that ND proteins play roles in the capacity of NAD(P)H oxidation (*nda1*) [21], modulating the total leaf NADPH/NADP + ratios (NDB1 over expressor) [43], reative oxygen species formation and salinity tolerance (*ndb4*) [19]. The functions of ND proteins have been proposed presuming an exclusive mitochondrial localization. However, as shown in this study, and other studies [1,44,45], the targeting ability of a protein depends on the test tissue and the constructs used, and cell specificity cannot be ruled out, i.e. dual targeting may occur in specific types of cells. Previous studies analyzing the location of NDB1 in potato tubers concluded that there was no NDB1 in peroxisomes [46,47]. This difference to this study may be due to several reasons, firstly, there is a difference between targeting and accumulation, and the specialized storage tissue of potato tubers may not be an ideal tissue to study the location of an enzyme, especially if stem/shoot phenotypes are being examined. Secondly there are a number of technical problems with the methods used. The method used to purify the peroxisomes from the mitochondria [48], is claimed to produce pure organelles with only 2% or less cross- contamination. However in both cases only catalase activity is used to verify organelle purity [46,47]. Subsequent proteomic studies in yeast, mammalian and plant systems show that mitochondrial contamination is the most abundant contaminant of peroxisomes and vice versa [49]. Also Catalase is present in even highly purified mitochondria [50]. This suggests that the immunological approaches used in previous studies were not quantitative or sensitive enough [46,47].

While various roles for ND protein in mitochondria and plastids have been ascribed as outlined above, no function(s) has been described in peroxsiomes. Type II ND proteins are able to oxidize both NADH and NADPH and both are produced in peroxisomes via Isocitrate dehydrogenase, Glucose-6-phosphate dehydrogenase and 6-phosphogluconate dehydrogenase. Malate dehydrogenase and Hydroxyacyl-CoA dehydrogenase can also produce NADH in plant peroxisomes [51]. While other enzymes can oxidise NADH and NADPH in peroxisomes, this is also true for mitochondria and plastids, that have several systems to oxidise NADH or NAD(P)H [52,53].

The nature of the acquisition of dual targeting to peroxisomes and mitochondria of plant ND proteins is unknown, but as it only requires the modification of the terminal 3 amino acids of a protein, it can be readily envisaged that it can occur. It can also be seen that not all the PTS1 sequences are the same in the different ND proteins from different species (Figure 1). This indicates that the last three amino acids are not conserved due to functional constraints. This is supported by the crystal structure of the yeast type II ND protein Ndi1, which illustrated that the C-terminal region is not required for enzymatic activity [54,55]. Rather, the C-terminal region is required for attaching Ndi1 to the inner mitochondrial membrane. This may mean that the C-terminal region of type II NDs may be able to readily undergo mutations which does not affect the catalytic activity of the protein. But may change the function as the protein is now targeted to an additional location, where it can carry out a new function.

The dual targeting of ND proteins in plants to mitochondria and peroxisomes is interesting because it involves two distinct targeting signals at opposite ends of the proteins. Examples of this have been previously reported to occur naturally, in Arabidopsis acyl-activating enzymes which display an identical arrangement of targeting signals except the mitochondrial signal is replaced with a chloroplast signal [56], and an artificially created protein containing the chloroplast targeting signal of Small subunit unit of rubisco (SSU) attached to both RFP and GFP followed by a PTS1 signal, could in fact target to both chloroplasts and peroxisomes *in vivo*[57]. This suggests that both signals can be recognized in the cell environment, even though the N-terminal signal would emerge from the ribosome before the C-terminal this differs slightly from previous work performed on the targeting signals of dual targeted proteins [58,59]. However these studies were focused on dual targeting between mitochondria and chloroplasts, where the targeting signals are both located at the N-terminus. Thus regulation of import or binding factors may occur, to distribute dual targeted proteins.

## Conclusions

**Figure 8:**
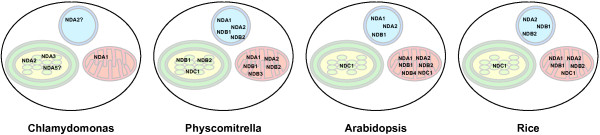
**Summary of the subcellular localizations of type II ND proteins in plants.** Combining the results from this study and other previous studies of the subcellular locations in four model plant species (*Chlamydomonas reinhardtii, Physcomitrella patens, Arabidopsis thaliana* and *Oryza sativa*) of the Type II ND proteins. Question marks denote predicted possible localizations. Chloroplasts - green, Mitochondria - red and Peroxisomes - blue.

In conclusion this study shows that ND proteins are targeted to multiple organelles in land plants (Figure 8). We assessed targeting from three major groups of plants, Dicots = Arabidopsis, a Moss = Physcomitrella, and a Monocot = rice, where we used Allium (onion). Thus targeting was assesses for all three species that belonged to that major plant group. This multiple targeting ability arose early in the evolution of land plants. It is important that the understanding of the location of protein(s) be taken from a wide variety of sources as possible. While models like Arabidopsis have provided much information, it should not be presumed that all plants are the same with respects to the location of orthologous proteins. Targeting signals of proteins are less constrained by specific sequence requirements than active sites of proteins, and therefore may be acquired and/or altered over time. While it is not feasible to interrogate the subcellular proteomes of a variety of plants species to the same degree as has been obtained in Arabidopsis, discounting evolutionary differences would mean that all plants should be the same, and by definition that is not the case.

## Methods

### Bioinformatic analyses

Protein sequences for all Arabidopsis type II NAD(P)H dehydrogenase proteins were obtained from the TAIR website (http://www.arabidopsis.org) (NDA1; At1g07180, NDA2; At2g29990. NDB1; At4g28220, NDB2; At4g05020, NDB3; At4g21490, NDB4; At2g20800, NDC1; At5g08740) and used in BlastP [60] searches against *Volvox carteri, Chlamydomonas reinhardtii* (Chlamydomonas), *Physcomitrella patens*, *Selaginella moellendorffii*, *Oryza sativa*, *Zea mays*, *Vitis vinifera*, *Glycine Max*, and *Populus trichocarpa* protein databases using the Phytozome website (http://www.phytozome.net). Blastx searches were used in order to identify proteins from *Picea glauca* on the NCBI database (http://www.ncbi.nlm.nih.gov) [60]. The known sequences for the *Escherichia coli*, *Saccharomyces cerevisiae*, and *Synechocystis sp. PCC 6803* NADH dehydrogenases were downloaded from the NCBI website (http://www.ncbi.nlm.nih.gov). Predictions of subcellular localizations were carried out using Predotar [61], TargetP [62] and Wolfpsort [63]. The ATP predictor [64] was also utilized to predict the probability of a given protein to be dual targeted to mitochondria and plastids. Peroxisomal targeting was predicted using the PTS1 predictor [29], PredPlantPTS1 [30] and by comparing the last 3 C-terminal amino acids with the known PTS1 sequences from the AraPerox database [31]. Phylogenetic analysis was carried out by first creating a multiple sequence alignment of all proteins using MAFFT [65]. The phylogenetic tree was calculated using MEGA 5 [66] using statistical method of neighboring-joining with the number of bootstrap replications of 1000.

### Cloning and constructing of vectors

RNA extraction from *Arabidopsis thaliana*, *Oryza sativa* and *Physcomitrella patens* was carried out using the RNeasy kit (Qiagen, Melbourne) according to manufacturer’s instructions. Reverse transcription was carried out using SuperScript™ III First-strand synthesis system (Invitrogen, Sydney). For some genes, cDNA clones were obtained from the Knowledge-based Oryza Molecular biological Encyclopedia (KOME) [67] or the RIKEN Bioresource Center [68]. cDNA or cDNA clones were used as templates to amplify full-length cDNAs or the last C-terminal 10 amino acids prior to the stop codon for cloning into N and C-terminal GFP vectors [3,25] using recombinant Gateway technology (Invitrogen, Sydney). The targeting signals of the *Pisum sativum* small subunit of 1,5 – ribulose bisphosphate carboxylase/oxygenase (SSU) and *Cucubita sp*. malate synthase were fused to RFP and used as a plastid and peroxisomal markers respectively [3,69]. For mitochondrial controls three different constructs were utilised. For Arabidopsis suspension cells the targeting signal of the *Glycine max* alternative oxidase (AOX) was used [3]. For onion cells the cytochrome c oxidase IV targeting signal from *Saccharomyces cerevisiae* fused to mCherry was utilised [70]. Finally the full length coding sequence from the Physcomitrella alternative oxidase (Pp1s183_11V6) fused to mCherry was used in Physcomitrella tissue [45].

### Subcellullar localizations

Biolistic co-transformation of GFP and RFP/mCherry fusion vectors was performed on Arabidopsis cell suspensions and onion epidermal cells as previously described [3]. Briefly, 5 μg of each GFP and RFP plasmid were co-precipitated onto gold particles and bombarded onto 4-day-old Arabidopsis cell suspensions and freshly peeled onion epidermal cells using the PDS-1000/He biolistic transformation system (Bio-Rad, Sydney). For putative Physcomitrella proteins, transformation was also performed on 7-day-old protonemal tissues. Following incubation for 12–24 h at 22°C (25°C for Physcomitrella) in the dark, GFP and RFP expression was visualized at 100X magnification using a BX61 Olympus microscope (Olympus, Melbourne) with the excitation wavelengths of 460/480 nm (GFP) and 535/555 nm (RFP), and emission wavelengths of 495–540 nm (GFP) and 570–625 nm (RFP). Images were captured using Cell^R^ imaging software (Olympus, Melbourne) as previously described [3].

## Competing interests

The authors declare that they have no competing interests.

## Authors’ contributions

LX performed the experimental work with the assistance of SRL and MM. CC and JW oversaw the analysis, design and implementation of the study. CC and JW drafted the manuscript. All authors read and approved the final manuscript.

## Supplementary Material

Additional file 1 **List of known dual targeted proteins in plants.** Indicated for each protein is its known locations, plant species, accession number and functional description. It is also indicated the method used to determine dual targeting. The proteomics column refers to if the protein has been identified in proteomic studies in both of its target organelles. Click here for file

Additional file 2 **Subcellular localizations of Arabidopsis, rice and Physcomitrella NDC type proteins.** Summary of subcellular localization data of plant NAD(P)H dehydrogenases. Indicated is which species and accession number for each protein. The predicted and confirmed subcellular localizations of each protein are indicated. Also shown are the known Peroxisomal type 1 (PTS1) targeting signals from plants which are ordered by the classification from the Araperox database. Blue colour indicates the PTS1 classification. M = mitochondrial, Pl = plastid and Px = peroxisomal. Click here for file

Additional file 3 **Multiple sequence alignment of the N-terminal regions of NDB proteins.** Shown is a multiple sequence alignment of the N-terminal regions for the *Chlamydomonas reinhardtii* (Cr), *Physcomitrella patens* (Pp), *Oryza sativa* (Os) and *Arabidopsis thaliana* (At) NDB protein sequences. Highlighted by the red boxes are the N-terminal extensions found in Chlamydomonas and Physcomitrella sequences. Click here for file
