# Supplementary material for: The dual targeting ability of type II NAD(P)H dehydrogenases arose early in land plant evolution
Source: BMC Plant Biol. 2013 Jul 10;13:100. doi: 10.1186/1471-2229-13-100 (PMC3716789; doi:10.1186/1471-2229-13-100)
Supplement: Additional file 3 — Multiple sequence alignment of the N-terminal regions of NDB proteins. Shown is a multiple sequence alignment of the N-terminal regions for the Chlamydomonas reinhardtii (Cr), Physcomitrella patens (Pp), Oryza sativa (Os) and Arabidopsis thaliana (At) NDB protein sequences. Highlighted by the red boxes are the N-terminal extensions found in Chlamydomonas and Physcomitrella sequences. [file 1471-2229-13-100-S3.pdf]

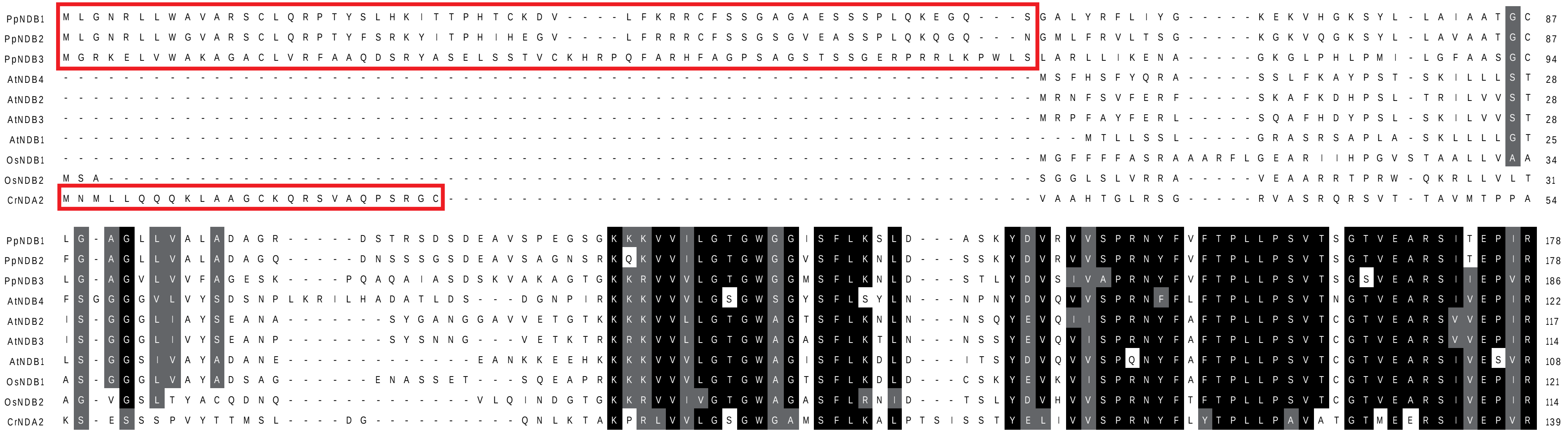
PpNDB1 MLGNRLLWAVARSCLQRPTYSLHKITTPHTCKDV----LFKRRCFSSGAGAESSSPLQKEGQ---SGALYRFLIYG-----KEKVHGKSYL-LAIAATGC 87
PpNDB2 MLGNRLLWGVARSCLQRPTYFSRKYITPHIHEGV----LFRRRCFSSGSGVEASSPLQKQGQ---NGMLFRVLTSG-----KGKVQGKSYL-LAVAATGC 87
PpNDB3 MGRKELVWAKAGACLVRFAAQDSRYASELSSTVCKHRPQFARHFAGPSAGSTSSGERPRRLKPWLSLARLLIKENA-----GKGLPHLPMI-LGFAASGC 94
AtNDB4 --MSFHSFYQRA-----SSLFKAYPST-SKILLLST 28
AtNDB2 --MRNFSVFERF-----SKAFKDHPSL-TRILVVST 28
AtNDB3 --MRPFAYFERL-----SQAFHDYPSL-SKILVVST 28
AtNDB1 ---MTLLSSL-----GRASRSAPLA-SKLLLLGT 25
OsNDB1 --MGFFFFASRAAARFLGEARIIHPGVSTAALLVAA 34
OsNDB2 MSA---SGGLSLVRRA-----VEAARRTPRW-QKRLLVLT 31
CrNDA2 MNMLLQQQKLAAGCKQRSVAQPSRGC--VAAHTGLRSG-----RVASRQRSVT-TAVMTPPA 54
PpNDB1 LG-AGLLVALADAGR-----DSTRSDSDEAVSPEGSGKKKVVILGTGWGGISFLKSLD---ASKYDVRVVSPRNYFVFTPLLPSVTSGTVEARSITEPIR 178
PpNDB2 FG-AGLLVALADAGQ-----DNSSSGSDEAVSAGNSRKQKVVILGTGWGGVSFLKNLD---SSKYDVRVVSPRNYFVFTPLLPSVTSGTVEARSITEPIR 178
PpNDB3 LG-AGVLVVFAGESK----PQAQAIASDSKVAKAGTGKKRVVVLGTGWGGMSFLKNLD---STLYDVSIVAPRNYFVFTPLLPSVTSGSVEARSIIEPVR 186
AtNDB4 FSGGGGVLVYSDSNPLKRILHADATLDS---DGNPIRKKKVVVLGSGWSGYSFLSYLN---NPNYDVQVVSPRNFFLFTPLLPSVTNGTVEARSIVEPIR 122
AtNDB2 IS-GGGLIAYSEANA-------SYGANGGAVVETGTKKKKVVLLGTGWAGTSFLKNLN---NSQYEVQIISPRNYFAFTPLLPSVTCGTVEARSVVEPIR 117
AtNDB3 IS-GGGLIVYSEANP-------SYSNNG---VETKTRKRKVVLLGTGWAGASFLKTLN---NSSYEVQVISPRNYFAFTPLLPSVTCGTVEARSVVEPIR 114
AtNDB1 LS-GGSIVAYADANE-------------EANKKEEHKKKKVVVLGTGWAGISFLKDLD---ITSYDVQVVSPQNYFAFTPLLPSVTCGTVEARSIVESVR 108
OsNDB1 AS-GGGLVAYADSAG------ENASSET---SQEAPRKKKVVVLGTGWAGTSFLKDLD---CSKYEVKVISPRNYFAFTPLLPSVTCGTVEARSIVEPIR 121
OsNDB2 AG-VGSLTYACQDNQ-------------VLQINDGTGKKRVVIVGTGWAGASFLRNID---TSLYDVHVVSPRNYFTFTPLLPSVTCGTVEARSIVEPIR 114
CrNDA2 KS-ESSSPVYTTMSL----DG----------QNLKTAKPRLVVLGSGWGAMSFLKALPTSISSTYELIVVSPRNYFLYTPLLPAVATGTMEERSIVEPVR 139
